# Oxygen–Ozone Therapy in Tendinopathy Management: A Comprehensive Review

**DOI:** 10.3390/jpm15100459

**Published:** 2025-09-30

**Authors:** Giacomo Farì, Giovanni Pignatelli, Sara Clelia Longo, Fabrizio Brindisino, Giuseppe Giovannico, Simone Della Tommasa, Laura Dell’Anna, Luisa De Palma, Francesco Quarta, Andrea Bernetti

**Affiliations:** 1 Department of Experimental Medicine (Di.Me.S.), University of Salento, 73100 Lecce, Italy; andrea.bernetti@unisalento.it; 2 Physical and Rehabilitation Medicine Unit, A. Perrino Hospital, ASL Brindisi, 72100 Brindisi, Italy; giovannipignatelli88@gmail.com; 3 Rehabilitation Unit, M. Paternò Arezzo Hospital, Provincial Health Authority, 97100 Ragusa, Italy; clelialongo29@gmail.com; 4 Department of Medicine and Health Science “Vincenzo Tiberio”, University of Molise c/o Cardarelli Hospital, C/da Tappino, 86100 Campobasso, Italy; fabrizio.brindisino@unimol.it (F.B.); giuseppe.giovannico@unimol.it (G.G.); 5 Department for Horses, University of Leipzig, 04103 Leipzig, Germany; della.tommasa@vetmed.uni-leipzig.de; 6 Department of Translational Biomedicine and Neuroscience (DiBraiN), Aldo Moro University, 70121 Bari, Italy; l.dellanna3@studenti.uniba.it (L.D.); luisa.depalma@policlinico.ba.it (L.D.P.); 7 Department of Biological and Environmental Science and Technologies (Di.S.Te.B.A.), University of Salento, 73100 Lecce, Italy; francesco.quarta6@studenti.unisalento.it; 8 Infradepartmental University Program of Physical and Rehabilitation Medicine, “V. Fazzi” Hospital, ASL Lecce, 73100 Lecce, Italy

**Keywords:** pain, tendon, tendinopathy, complementary therapy, oxidative stress, injections

## Abstract

**Background**: Tendinopathy is a degenerative condition caused by mechanical overload, accounting for approximately 30% of musculoskeletal healthcare cases. It progresses through a process characterized by collagen disorganization, altered vascularization, and neuronal ingrowth. Traditional conservative treatments, such as therapeutic exercises, non-steroidal anti-inflammatory drugs, and physical therapies, are useful, but their effectiveness is sometimes only partial and there is a need to search for new potential solutions. Recent interest in oxygen–ozone (O_2_-O_3_) therapy stems from preliminary observations suggesting potential anti-inflammatory and regenerative effects. Nevertheless, its clinical role remains speculative and warrants thorough investigation beyond anecdotal evidence. Considering the heterogeneity of clinical presentations and treatment responses among patients, O_2_-O_3_ therapy has been proposed as a promising tool for tailoring personalized treatment strategies for tendinopathy. This review critically appraises the available literature concerning the mechanistic rationale and clinical applications of O_2_-O_3_ therapy in tendinopathy, with attention to both its theoretical underpinnings and the quality of empirical evidence. **Methods**: A literature search was conducted on O_2_-O_3_ therapy for tendinopathy using PubMed, Cochrane, and Embase, filtering for full-text articles published between 2004 and 2024. Recent clinical trials were included irrespective of evidence level, while excluding systematic reviews, duplicates, and irrelevant studies. **Results**: Ozone has been shown to modulate oxidative stress, promote neovascularization, and suppress pro-inflammatory cytokines. Both clinical and in vivo studies indicate that O_2_-O_3_ therapy relieves pain, enhances tendon healing, and improves biomechanical properties. Some comparative studies suggest that O_2_-O_3_ therapy might provide more sustained symptoms control than corticosteroids, but the heterogeneity of follow-up durations and outcome measures prevents definitive conclusions. **Conclusions**: O_2_-O_3_ therapy emerges as a potentially valuable adjunct in the management of chronic tendinopathy, particularly in cases unresponsive to conventional treatments. However, its clinical role remains to be clearly defined and its possible role in personalized medicine needs further exploration, particularly in relation to patient stratification and individualized treatment protocols. Further high-quality randomized controlled trials are warranted to validate its efficacy, determine long-term outcomes, and standardize treatment protocols to ensure safety and reproducibility.

## 1. Introduction

Tendons are fibrous connective tissues that connect muscles to bones, allowing the transmission of the force necessary for movement [1]. Their biomechanical properties provide tensile strength and elasticity, assisting muscle contraction and joint motion. Tendon structure extends throughout the muscle, not just at the enthesis, where connective tissue layers (epimysium, perimysium, and endomysium) merge near the bone surface [2]. Tendon morphology varies with muscle function; powerful muscles have shorter, broader tendons, while finer movement muscles have longer, thinner tendons [3]. Tendons and muscles represent a single functional unit. Microscopically, tendons consist of specialized cells and an extracellular matrix (ECM). Tenocytes and tenoblasts make up 90–95% of tendon cells, while the rest include chondrocytes, synovial cells, and vascular cells [4]. Tenoblasts mature into tenocytes, which maintain ECM turnover and adapt to mechanical loads, communicating via gap junctions [5]. Gap junctions allow metabolite and ion exchange, regulating tendon adaptation through collagen synthesis modulation [6]. Tenocytes produce ECM components like collagen and proteoglycans. Type I collagen, making up 65–80% of the ECM, provides tensile strength, while proteoglycans contribute to viscoelasticity [7]. Type II collagen is found in fibrocartilaginous areas of the osteotendinous junction, with elastin, proteoglycans, and glycoproteins constituting 4%, 4%, and 2% of the ECM, respectively. Collagen fiber orientation influences tendon mechanics. Tendons have a hierarchical structure: tropocollagen molecules form microfibrils, and then fibrils, fibers, bundles, and fascicles. Fascicle diameter peaks between ages 20–29 but decreases with aging or excessive loading, potentially reducing muscle strength [3]. Tendon vascularization has intrinsic and extrinsic sources. Intrinsic vascularization occurs at the myotendinous junction, nourishing the proximal third of the tendon. The central portion receives extrinsic vascularization via the paratenon or synovial sheath [8]. This arrangement contributes to tendon pathology and rupture sites [9]. Tendons transmit muscular forces to bones, generating movement and absorbing external forces to prevent muscle damage. Their composition varies across anatomical sites, adapting stiffness and elasticity to mechanical demands. Strain rate influences tendon properties through collagen behavior and cross-linking patterns. At rest, collagen fibers are crimped. Under 2% strain, they straighten into a parallel structure. If strain stays below 4%, fibers return to their resting state, but strains exceeding 8–10% cause microscopic damage [10].

Tendinopathy refers to a pathological condition of tendons caused by excessive mechanical load or energy application. It accounts for approximately 30% of musculoskeletal healthcare visits [11]. The condition is characterized by pain, functional limitation, swelling, and inflammation, significantly impacting patients’ quality of life. In severe cases, it may lead to work restrictions, affecting socioeconomic status [12]. Historically, tendinopathy was classified as tendinitis, assuming inflammation as the primary pathological mechanism. However, research has shown tendon degeneration without significant inflammatory cell infiltration, leading to a broader definition encompassing failed healing responses, tenocyte proliferation, collagen fiber disruption, and increased non-collagenous matrix production [13,14,15]. This has led to a paradigm shift in understanding tendon pathology. Cook and Purdam proposed a continuum model of tendinopathy, consisting of three progressive stages: reactive tendinopathy, tendon disrepair, and degenerative tendinopathy [16]. The reactive phase results from acute overload, causing non-inflammatory cellular and matrix proliferation. This phase is reversible if the mechanical load is appropriately managed. If overload persists, the tendon enters the disrepair phase, characterized by disrupted collagen fibers, increased vascularization, and neuronal ingrowth, which can be detected via imaging techniques such as Magnetic Resonance Imaging (MRI) and ultrasound. The final degenerative stage involves irreversible cellular apoptosis and matrix disorganization, often presenting with thickened tendons and nodular formations [16]. Tendinopathy prevalence varies based on age, gender, and anatomical site. It is less frequent in younger individuals due to greater tissue elasticity but increases with age. Some evidence suggests a higher male predisposition, though this trend may change with increased female participation in sports [17]. In younger individuals, tendinopathies commonly affect the entheses, as seen in Osgood–Schlatter and Sever’s diseases [18]. Risk factors include age, sex, heavy physical labor, training volume, obesity, hormone replacement therapy, diabetes, fluoroquinolone use, and biomechanical imbalances. Although comprehensive epidemiological data encompassing all variables are lacking, tendinopathy remains a prevalent condition with substantial functional and quality of life impacts, leading to high therapeutic demand.

The classic presentation of tendinopathy involves progressive pain at the affected tendon site, often associated with increased activity. Pain is typically load-dependent. In early-stage tendinopathy, pain may appear at the beginning of an activity, subside during the activity, and reappear with prolonged exertion, eventually leading to functional impairment. Patients can usually pinpoint the pain location, describing it as “sharp” or “acute” in the initial phases and later as a “dull” and constant ache that may become acute again with specific movements [19].

Diagnosis is primarily clinical, supported by imaging modalities such as ultrasound and, in some cases, MRI. A thorough physical examination is required, assessing muscle atrophy, asymmetry, swelling, and erythema. Atrophy is often indicative of chronic conditions and provides insight into the duration of tendinopathy. Swelling, erythema, and asymmetry are commonly observed in pathological tendons. Range of motion (ROM) assessment of the affected joint is crucial, as ROM is often restricted on the symptomatic side. Specific tests that overload the tendon to reproduce pain, such as the Finkelstein test or Jobe test are also used [20]. Imaging is considered secondary to clinical evaluation. Traditionally, ultrasound and MRI have been regarded as the “gold standard” for diagnosing structural abnormalities and confirming clinical suspicions. The main diagnostic criteria include detecting structural abnormalities that correlate with positive clinical findings. However, the role of imaging in diagnosing conditions such as rotator cuff tendinopathy remains debated, as recent studies indicate tendon morpho-structural changes may be present even in asymptomatic individuals [21]. In recent years, ozone therapy has gained increasing attention as an adjuvant treatment for tendinopathies, owing to its anti-inflammatory, analgesic, and regenerative properties. Unlike more traditional pharmacological approaches, such as corticosteroids, medical ozone acts by modulating oxidative stress and promoting the release of anti-inflammatory cytokines, thereby exerting a positive effect on the damaged tendinous microenvironment. Experimental studies have demonstrated that local infiltrations of an oxygen–ozone (O_2_–O_3_) mixture can stimulate collagen regeneration, enhance local vascularization, and reduce the production of pro-inflammatory mediators such as IL-1β and TNF-α [22], offering a physiological alternative to corticosteroids and non-steroidal anti-inflammatory drugs (NSAIDs) in the conservative management of tendinopathies [23].

Recent evidence suggests that the combined use of invasive therapies with therapeutic exercise may provide additional benefits in the management of tendinopathy. Exercise plays a fundamental role, as it not only modulates pain perception but also delivers a mechanical loading stimulus that supports tendon remodeling. Specifically, it promotes longitudinal collagen alignment, increases tendon cross-sectional area, and contributes to muscle strengthening and enhanced resistance capacity of the tendon. In this context, the integration of ozone therapy or other invasive techniques with structured exercise programs could represent a promising strategy to optimize functional recovery [24].

Within this framework, and considering its regenerative potential, O_2_-O_3_ therapy represents a promising therapeutic option to be integrated into a multidisciplinary conservative management strategy for tendinopathies. Although several studies have explored the role of O_2_-O_3_ therapy in tendinopathy, the current scientific literature is fragmented and lacks methodological rigor. Nevertheless, the use of O_2_-O_3_ therapy could align with the principles of personalized medicine, offering the opportunity to tailor treatment strategies based on individual patient characteristics, such as inflammatory profiles, anatomical site, type and duration of symptoms, and response to different treatments. In light of the wide heterogeneity among patients and the variability in tendinopathy, a tailored and precise approach may reduce unnecessary interventions and optimize clinical outcomes. However, despite growing interest, the clinical efficacy of this approach remains insufficiently supported by robust, large-scale trials, underscoring the need for critical evaluation and synthesis of available data. Thus, the aim of this comprehensive review is to investigate the available literature concerning the scientific rationale and clinical applications of O_2_-O_3_ therapy in tendinopathy, with particular focus on its safety and potential efficacy.

## 2. Materials and Methods

Three independent authors conducted the article search. The electronic search engines used were PubMed (https://pubmed.ncbi.nlm.nih.gov, accessed on 16 February 2025), Cochrane (https://www.cochranelibrary.com, accessed on 16 February 2025), and Embase (https://www.embase.com accessed on 16 February 2025). The search terms were (“Oxygen-Ozone Therapy” OR “Ozone therapy” OR “Oxygen therapy”) AND (“Tendinopathy” OR “Tendon injury” OR “Tendonitis” OR “Tendon disorders”) AND (“Management” OR “Treatment” OR “Rehabilitation”).

**Figure 1:**
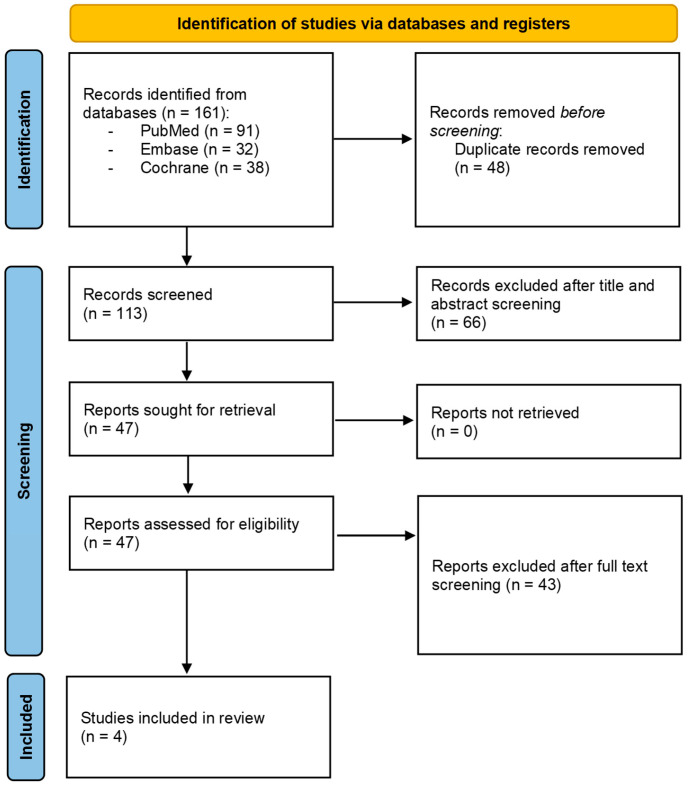
PRISMA flow chart of analyzed studies.

Then, the following filters were activated: text availability: full text; species: humans or animals; languages: English; and period: from 2004 to 2024. The references of the articles were manually examined to find the more relevant publications. Once the potential articles were gathered, they were further filtered based on specific criteria for inclusion or exclusion. The inclusion criteria were the following: articles published between 2004 and 2024 related to O_2_-O_3_ therapy in tendinopathy management; clinical trials and randomized trials; articles published in English. The exclusion criteria were the following: reviews, conference papers, study protocols, case reports, case series; articles published before 2004; articles related to manipulative therapy for other diseases treatment (Figure 1).

Three investigators independently assessed each title, abstract, and full-text article for the eligible studies and disagreements were resolved through discussion with two other investigators. All authors agreed on the final inclusion list.

## 3. Results

The studies were identified through a search on four databases (PubMed, Embase, and Cochrane). At the end of the selection process, 161 articles were extracted, of which 91 from PubMed, 32 from Embase and 38 from Cochrane. Then, duplicates have been eliminated (*n* = 48). After titles and abstracts screening, 66 articles were excluded. The full text of the remaining 47 studies was assessed; then, 43 articles were eliminated since they did not meet the inclusion criteria. Finally, four articles were included [25,26,27,28] (Figure 1).

Characteristics of the included studies are presented in Table 1. Ulusoy et al. [25] conducted a retrospective study involving 80 patients with chronic lateral epicondylitis, comparing the analgesic efficacy of corticosteroid injections with medical ozone therapy. Patients were evaluated using the modified Verhaar scoring system at baseline and at 3, 6, and 9 months post-injection. While both groups showed initial pain improvement, the ozone group demonstrated significantly superior outcomes at all follow-up points. At 3, 6, and 9 months, patients treated with ozone reported higher rates of “excellent” or “good” outcomes for pain at rest, under compression, and during activity (*p* < 0.001 for all comparisons). Specifically, by the 9-month follow-up, 92.8% of patients in the ozone group reported favorable outcomes for resting pain, compared to 39.5% in the corticosteroid group. Similar advantages were observed for pain under compression (88.1% vs. 34.2%) and during activity (85.7% vs. 34.3%). No clinically relevant adverse effects were reported in either group. Despite these promising findings, the authors acknowledged key limitations, including the retrospective design and lack of a control group, emphasizing the need for further prospective randomized controlled trials. In the study by Kizilkaya et al. [26], a histopathological and biomechanical assessment of Achilles tendons was conducted in an animal model to compare the effects of ozone therapy with a control intervention. By the second week, the ozone-treated group showed greater fibroblast proliferation (*p* = 0.042) and significantly reduced inflammation (*p* = 0.001). At weeks 4 and 6, the ozone group exhibited enhanced tissue remodeling and fibroblast activity, along with improved biomechanical properties, including greater tensile strength and higher failure load (*p* < 0.01). In a prospective, single-center randomized clinical trial by Atar et al. [27], 44 participants with chronic supraspinatus tendinopathy were randomly assigned to receive either corticosteroid injections or O_2_-O_3_ therapy. Clinical outcomes were measured using three validated scales: the VAS for pain, the WORC for quality of life, and the SPADI for shoulder function and disability. Repeated-measures analysis revealed a significant time effect on all outcome measures in both groups. Comparisons of baseline scores with those at 4 and 12 weeks indicated both clinically and statistically significant improvements over time, regardless of the intervention administered. In the randomized controlled trial conducted by Babaei-Ghazani et al. [28] involving 30 patients with shoulder impingement syndrome, ultrasound-guided injections of either ozone or corticosteroids were compared. Both groups demonstrated significant improvements in pain and function (VAS, SPADI, Constant score; *p* < 0.001), with the corticosteroid group showing superior short-term outcomes (*p* < 0.001). However, over time, pain continued to improve in the ozone group, whereas it slightly worsened in the corticosteroid group. No significant differences were found in ROM or ultrasonographic findings.

## 4. Discussion

### 4.1. Current Treatment Options for Tendinopathy: Scientific Evidence

Treatment selection should be tailored based on severity, compliance, pain levels, and symptom duration. Therapeutic options are categorized as conservative (pharmacological, physical therapy, rehabilitation), minimally invasive (peritendinous injections, percutaneous electrolysis, dry needling), and surgical approaches. Early-phase management involves functional rest of the affected tendon, with or without bracing to support tendon recovery. If symptoms persist, rehabilitation protocols incorporating targeted exercises are recommended. Eccentric training, particularly heavy eccentric loading based on the Alfredson protocol, is considered the gold standard [29]. However, evidence does not conclusively support eccentric exercise over other exercise modalities for pain and function improvement in tendinopathy. Some studies suggest eccentric exercises may be ineffective for athletes during competitive seasons, while isometric and resistance exercises have also shown potential benefits [30,31]. Exercise-induced hypoalgesia is a common response in healthy individuals, but central sensitization may impair this response in certain patients. Since pain relief is independent of exercise type, the distinction between isometric and eccentric exercises for pain management in tendinopathy becomes less relevant. In more complex cases, a combined approach incorporating rest, exercise, and NSAID therapy is often recommended. However, evidence supporting NSAID efficacy is of low quality, and their use is associated with significant gastrointestinal, cardiovascular, and renal side effects [32]. Given that many tendinopathy patients are elderly and on multiple medications, NSAID interactions, particularly with COX inhibitors like aspirin, must be considered, making these drugs less manageable [33]. Technological advancements have introduced physical therapies as therapeutic options for tendinopathy. Among these, extracorporeal shockwave therapy (ESWT) is the most extensively studied. ESWT is a mechanotherapy that stimulates cellular metabolism and acts directly on nociceptors, reducing pain. Acoustic shockwave signals are converted into biological signals that trigger cellular proliferation and differentiation through mechanotransduction. Most ESWT research focuses on elucidating the mechanosensitive feedback mechanisms between acoustic pulses and target cells, particularly ECM binding proteins and cytoskeletal components. However, the exact processes by which tissues recognize and convert acoustic signal parameters into biological responses remain unclear [34]. The analgesic effects of ESWT are also not fully understood but may involve modulation of primary afferent nociceptive C fibers [35]. Laser therapy, another common physical modality, encompasses a broad spectrum of wavelengths with varying frequencies and energy outputs. Evidence suggests laser therapy may promote tissue regeneration and increase local blood flow via vasodilation. However, there is no consensus on the optimal laser technology or its actual regenerative potential for tendinopathy [36]. Ultrasound therapy is frequently used for tendinopathy, though clinical evidence supporting its effectiveness remains limited. Most in vivo studies report variable efficacy, but methodological flaws and biases limit their validity. While RCTs on ultrasound therapy are scarce, some evidence suggests mild benefits in treating lateral epicondylitis and calcific supraspinatus tendinopathy [37].

A more recent physical therapy applied to tendinopathy is hyperthermia, which has shown promising preliminary results. In randomized controlled trials, hyperthermia has been compared to therapeutic ultrasound, with findings indicating superior pain relief and higher patient satisfaction in the hyperthermia group [38]. Other common interventions include iontophoresis and phonophoresis, which utilize ionizing currents or ultrasound to deliver drugs locally. Corticosteroids and NSAIDs are frequently administered through these methods. While widely used and anecdotally effective, well-designed RCTs are lacking to support reliable recommendations [21].

Current therapeutic strategies focus on managing pain, promoting tissue healing, and minimizing adverse effects. Pharmacological treatments, such as NSAIDs and corticosteroids, are limited by risks of drug interactions, gastrointestinal toxicity, and complications related to hypertension and diabetes. These agents are unsuitable for long-term use and lack tissue reparative properties; corticosteroids, in particular, can harm tendinous tissue. Physical therapies, while beneficial, are often expensive, less accessible, and contraindicated in patients with cancer, arrhythmias, or pacemakers.

### 4.2. Ozone Therapy

Ozone therapy, introduced as a medical treatment in the 19th century, gained prominence with Nikola Tesla’s patent for the first ozone generator [39]. The development of certified ozone generators has enabled precise preparation of O_2_-O_3_ mixtures, reducing toxicity risks from the high reactivity of ozone molecules [40]. Ozone (O_3_) exhibits notable medical properties, including bactericidal, virucidal, anti-inflammatory, and circulatory-stimulating effects, making it valuable for wound healing, vascularization, infection management, and treating chronic inflammatory conditions, particularly musculoskeletal disorders [41]. As one of the most potent natural oxidizing agents, ozone decomposes into oxygen at high concentrations. In human tissues, O_3_ reacts with water and polyunsaturated fatty acids (PUFAs) to form hydrogen peroxide (H_2_O_2_) and lipid oxidation products (LOPs) such as 4-HNE (from omega-6 PUFAs) and 4-HHE (from omega-3 PUFAs) [42]. H_2_O_2_ serves as a primary carrier of ozone, while other reactive oxygen species (ROS), including superoxide and hydroxyl radicals, are also generated [43]. ROS, once considered purely harmful, are now recognized as mediators of host defense and immune responses, with key roles in signal transduction. Endogenous scavengers, including superoxide dismutase, glutathione peroxidase, catalase, and NADPH quinone oxidoreductase, counteract moderate oxidative stress [44]. Repeated mild oxidative stress activates the transcription factor Nrf2, which dissociates from its inhibitory complex with Keap-1, translocates to the nucleus, and promotes the transcription of antioxidant response elements (AREs) [43,45,46]. This enhances cellular resistance to pathological oxidative stress, a hallmark of chronic inflammatory diseases [47]. Nrf2 activation also attenuates NF-κB signaling, reducing inflammation and muscle atrophy [48]. At low doses, O_3_ may suppress pro-inflammatory cytokines (e.g., IL-6, IL-8, TNF-α), regulate prostaglandin synthesis, and enhance macrophage and leukocyte activity, highlighting its potential to modulate inflammation [49].

### 4.3. Clinical Evidence Supporting O_2_-O_3_ Therapy

O_2_-O_3_ therapy has been investigated for its effects on musculoskeletal conditions, particularly in managing pain and functional limitations [50]. Pain, often associated with inflammation, may be alleviated by O_2_-O_3_ through increased production of serotonin and endogenous opioids, which stimulate anti-nociceptive pathways [51]. Additionally, tendinopathies are characterized by poor vascularization and hypoxia, impairing tissue repair. O_2_-O_3_ therapy can enhance nitric oxide, adenosine, and prostaglandin production in hypoxic tissues, promoting vasodilation, oxygen delivery, and the availability of growth factors, cells, and cytokines essential for repair [52]. These findings suggest that O_2_-O_3_ therapy may positively influence tendinopathies by addressing both pain and tissue regeneration, though its efficacy depends on managing the balance between beneficial and potentially toxic oxidative stress.

The increasing number of publications on O_2_-O_3_ therapy reflects a growing interest in its potential for treating chronic inflammatory disorders. Nonetheless, recognition within mainstream clinical practice remains limited due to the lack of consensus guidelines and insufficient high-level evidence. Studies suggest its efficacy in managing degenerative spine diseases, including low back pain, disk herniation, protrusions, and failed back surgery syndrome [53]. O_2_-O_3_ therapy has also shown promise in pain management for knee osteoarthritis, as highlighted by a systematic review by Sconza et al. [54].

Recent investigations have explored its application in tendon-related disorders. A randomized controlled trial demonstrated its potential in treating shoulder impingement [28], while Ulusoy et al. [25] reported favorable outcomes in chronic lateral epicondylitis unresponsive to conventional treatments. A study comparing corticosteroid and O_2_-O_3_ injections found both effective in reducing pain, but O_2_-O_3_ provided superior pain relief at 3, 6, and 9 months post-treatment, highlighting its potential for chronic lateral epicondylitis. In vivo studies support these clinical findings. Tendons treated with O_2_-O_3_ have shown improved collagen fiber organization and enhanced biomechanical strength, accompanied by reduced fibrosis and increased collagen synthesis [43]. Ozone therapy also accelerated tendon repair, reducing necrosis and improving tissue integration. Anti-inflammatory effects were evident, with reduced neutrophil and macrophage infiltrates and lower inflammatory protein levels. A 2018 study showed that O_2_-O_3_ promoted neovascularization in damaged tendons, increasing vascular density as confirmed by CD31 staining and immunohistochemical analysis [26]. Although recent studies report favorable outcomes with O_2_-O_3_ therapy in chronic tendinopathies, these findings must be interpreted with caution given the variability in methodologies and often limited sample sizes. Patients reported significant improvements in pain and function, with faster effects in acute cases and sustained benefits in chronic conditions. A 2023 review by Arias-Vázquez et al. demonstrated that O_2_-O_3_ injections reduce pain comparably or more effectively than NSAIDs [55]. For rotator cuff tendinopathy, O_2_-O_3_ therapy reduced pain and lesion size, as shown by ultrasound analysis [27]. Preliminary findings suggest similar efficacy in Achilles tendinopathy, with pain reduction, improved tendon healing, and decreased fibrosis.

O_2_-O_3_ treatments appear particularly effective in reducing pain and improving function in patients with chronic tendinopathies, such as rotator cuff tendinopathy and lateral epicondylitis. Patients treated with ozone report significant improvements in pain and functional scores as early as the first weeks of treatment. Injecting ozone directly into the lesion site reduces pain as effectively as, or even more effectively than, conventional treatments such as NSAIDs [55]. The therapeutic effect seems to occur more rapidly in cases of acute tendinopathy, whereas in chronic cases, more durable symptom relief and improved healing have been reported. Moreover, the application of ozone therapy in rotator cuff tendinopathy has yielded clearly positive results: patients receiving ozone injections demonstrated significant improvement in both pain and functional capacity compared to the control group, with ultrasound analysis showing a reduction in tendon lesion size [27]. Although preliminary, data on the use of ozone therapy in Achilles tendinopathies also suggest promising potential for pain reduction and enhanced fiber healing with less fibrosis. To be considered in the interpretation of the data is the fact that differences in outcomes likely reflect anatomical, pathological, and methodological variability. O_2_-O_3_ efficacy appears to be protocol- and site-dependent: multi-session regimens [25,27] yielded effects comparable to or superior to corticosteroids, whereas single-shot trials [28] favored steroids. These findings underscore the need for standardized RCTs harmonizing dose, frequency, target tissue, and co-interventions to better define the role of ozone therapy in upper-limb tendinopathies. Furthermore, the effects of oxygen–ozone therapy on chronic tendon injuries may be comparable to those observed in in vivo studies of bone tissue affected by osteoporosis. Osteoporosis induces biochemical changes in bone associated with alterations in local nerve fiber endings [56]. De Lima Neto et al. reported that ozone enhances bisphosphonate-induced bone regeneration [57]. In particular, treated bones demonstrated improved elasticity and dynamic strength, qualities that are also essential for the recovery of tendons affected by chronic degeneration.

The combination of ozone therapy with other rehabilitative techniques, especially therapeutic exercise, may represent a future avenue of research. While current evidence mainly addresses ozone therapy as a standalone treatment, integrating it with structured exercise programs could enhance functional recovery and clinical outcomes in tendinopathy. Further studies are needed to define possible treatment guidelines tailored to specific anatomical sites.

### 4.4. Safety Profile and Limitations of O_2_-O_3_ Therapy

As with all medical therapies, O_2_-O_3_ therapy presents contraindications and potential side effects that must be carefully considered when selecting the most appropriate treatment. The primary contraindications are related to the oxidative stress induced by ozone (O_3_) [58]. The main absolute contraindication is glucose-6-phosphate dehydrogenase (G6PD) deficiency, due to the risk of hemolysis caused by oxidative stress. Other contraindications include pregnancy (although considered relative), uncontrolled hyperthyroidism, severe cardiovascular diseases, and heart failure. O_2_-O_3_ concentrations should be maintained within a specific therapeutic range to ensure safety.

Despite appropriate precautions, patients may experience a transient sensation of heaviness at the injection site, which usually resolves spontaneously within minutes. Other adverse effects may occur due to improper administration techniques, such as vasovagal reactions, pain, hematoma, local infections, and in rare cases, fatal complications [59]. To minimize risks, ultrasound guidance has been proposed as a non-invasive technique that allows real-time visualization of soft tissues, facilitates precise injection of the O_2_-O_3_ mixture, and enables monitoring of gas diffusion, thereby reducing the risk of adverse events [60].

Studies indicate that oxygen–ozone therapy is generally well tolerated and associated with minimal side effects. Further supporting its clinical utility, De Sire et al. highlighted in a 2022 review the low incidence of adverse events associated with O_2_-O_3_ therapy and its capacity to reduce systemic inflammatory markers, such as IL-6 and TNF-α. These findings contribute to a growing consensus on the therapy’s safety when administered using controlled protocols [61]. The most commonly reported adverse effects include mild burning or irritation at the injection site, which typically resolves quickly. Systemic side effects, although uncommon, may include headache, fatigue, dizziness, or nausea, particularly when ozone is administered systemically. Allergic reactions are rare but can manifest as symptoms such as rash, itching, or swelling, with anaphylaxis being extremely rare. Severe complications are highly uncommon, especially when the procedure is performed by trained professionals. However, potential risks include gas embolism, which may occur if ozone is inadvertently injected into the bloodstream, though this is exceedingly rare with proper technique. Improper injection or excessive ozone dosage may result in nerve or tissue injury, as well as muscle damage if the procedure is performed incorrectly or too frequently. Given these risks, oxygen–ozone therapy is contraindicated or should be used with caution in specific clinical conditions. Its use during pregnancy is generally discouraged due to insufficient safety data. It is also not recommended for patients with severe cardiovascular conditions, such as recent myocardial infarction or heart failure, as ozone administration may affect blood pressure and increase cardiac workload. Furthermore, in patients with hemoglobinopathies, ozone may interfere with oxygen transport, potentially exacerbating hypoxic states [43].

## 5. Conclusions

Recent scientific evidence suggests that O_2_-O_3_ therapy is a promising therapeutic option for tendinopathies, showing favorable outcomes in terms of pain reduction, improved tendon function, and accelerated healing. The benefits appear particularly evident in cases of chronic tendinopathies that are unresponsive to conventional treatments such as anti-inflammatory drugs or physical therapy. Therefore, ozone therapy may also be employed as a complement to physiotherapy, promoting a faster and more sustained recovery. Compared to traditional approaches such as analgesics or corticosteroid injections, ozone therapy offers the advantage of fewer long-term side effects, reducing, for instance, the risk of tendon damage associated with repeated corticosteroid use or the systemic adverse effects of NSAIDs and analgesics. Moreover, ozone therapy has been shown to stimulate the body’s natural healing mechanisms, contributing to a more physiological and less invasive reparative process. As a potent oxidizing agent, when administered in a peritendinous manner, ozone reduces inflammation, enhances blood perfusion, and stimulates the production of endogenous antioxidants such as glutathione. This promotes tissue regeneration and alleviates pain, a hallmark of tendinopathies. In this context, O_2_-O_3_ therapy may represent an interesting addition to the range of personalized conservative treatments, in particular in patients with chronic conditions of specific inflammatory profiles or resistance to standard therapies.

Despite these promising results, further studies involving larger sample sizes and longer follow-up periods are necessary to confirm the long-term effects of ozone therapy on tendon structure and recurrence prevention. To improve clinical applicability, future research should prioritize the development of standardized protocols regarding ozone dosage, administration frequency, and target populations, as current variability undermines reproducibility and hinders clinical translation. Moreover, the integration of stratification criteria and comorbidities may enhance the identification of patients subgroups who could benefit from O_2_-O_3_ therapy. This would strengthen the role of this therapy in personalized rehabilitation programs.

In the context of chronic tendinopathies refractory to standard treatment, ozone therapy may offer a novel therapeutic avenue worth exploring. However, its role should still be considered experimental until additional evidence confirms its efficacy and safety across diverse clinical scenarios. Unlike conventional therapies that primarily target symptom relief, O_2_-O_3_ therapy may exert a dual effect by modulating nociceptive pathways and promoting tissue remodeling. Nevertheless, these proposed benefits require validation through mechanistic studies and direct comparisons with established interventions. To enhance clinical translation, future investigations must address current inconsistencies in administration protocols and incorporate long-term follow-up to determine the durability and reproducibility of therapeutic outcomes.

## Figures and Tables

**Table 1:** Summary of the selected studies.

Title	First Author and Publication Year	Study Sample and Design	Outcomes of the Study	Results
Comparison of corticosteroid injection and ozone injection for relief of pain in chronic lateral epicondylitis	Ulusoy G.R. et al., 2019 [25]	Retrospective cohort study. 80 patients with unilateral chronic lateral epicondylitis. One group treated with ozone injections (*n* = 42) and the other group treated with a single corticosteroid injection (*n* = 38). No co-interventions or activity restrictions.	Pain associated with chronic lateral epicondylitis assessed with Verhaar scores. Outcomes assessed at baseline, 3, 6, and 9 months.	The ozone therapy group showed significantly better pain scores compared to the corticosteroid group at 3, 6, and 9 months after injection.
Effectiveness of Ozone Therapy on Tendon Healing: An Experimental Study in Generated Achilles Tendon Injury Model in Rats	Kizilkaya, V. et al., (2018) [26]	Experimental study. 60 male Wistar rats. Rats were randomly assigned to the ozone therapy group (*n* = 30) or to the no treatment group (*n* = 30).	Remodeling, proliferation, collagen deposition, inflammation. Tendon tissues were harvested at 2, 4, and 6 weeks post-injury for histopathological and biomechanical analysis.	The ozone therapy had beneficial effects on Achilles tendon rupture healing.
Comparison of ultrasound-guided subacromial corticosteroid and ozone injections in the treatment of chronic rotator cuff tendinopathy: a randomized clinical trial	Atar MÖ. et al., (2023) [27]	Randomized clinical trial. 44 patients with chronic supraspinatus tendinopathy randomly assigned to the ozone group (*n* = 22) or corticosteroid group (*n* = 22). Injections in both groups were administered into subacromial bursa with an ultrasound-guided in-plane posterolateral approach. Both groups followed a daily shoulder exercise program.	Primary outcome measure was the change in the Western Ontario Rotator Cuff Index (WORC) score. Secondary outcome measures included Visual Analog Scale (VAS) and Shoulder Pain and Disability Index (SPADI) scores. Outcomes were assessed at 4 and 12 weeks.	Both the groups showed clinically significant improvements in shoulder pain, quality of life, and function
A Randomized Control Trial of Comparing Ultrasound-Guided Ozone vs. Corticosteroid Injection in Patients With Shoulder Impingement.	Babaei-Ghazani A. et al., (2019) [28]	Randomized control trial. 30 patients with shoulder impingement were randomly assigned to the ultrasound-guided injection with ozone group (*n* = 15) or ultrasound-guided injection with corticosteroid (*n* = 15). Both groups followed a daily home-based physical therapy program	Shoulder pain, disability scale, shoulder ROM, ultrasonographic measures. Outcomes were assessed at 2 and 8 weeks.	Both groups showed significant improvements in VAS, Shoulder Pain and Disability Scale. No statistical differences were observed between the groups in terms of ROM and ultrasonographic measures.

## Data Availability

Not applicable.

## Abbreviations

The following abbreviations are used in this manuscript:

O_2_-O_3_	Oxygen–ozone
NSAIDs	Non-steroidal anti-inflammatory drugs
ECM	Extracellular matrix
ROM	Range of motion
MRI	Magnetic Resonance Imaging
VAS	Visual Analog Scale
WORC	Western Ontario Rotator Cuff Index
SPADI	Shoulder Pain and Disability Index
ESWT	Extracorporeal shockwave therapy
PUFAs	Polyunsaturated fatty acids
H_2_O_2_	Hydrogen peroxide
LOPs	Lipid oxidation products
ROS	Reactive oxygen species
AREs	Antioxidant response elements
